# Diversity of *Yersinia enterocolitica* isolated from pigs in a French slaughterhouse over 2 years

**DOI:** 10.1002/mbo3.751

**Published:** 2018-10-22

**Authors:** Pierre Raymond, Emmanuelle Houard, Martine Denis, Emilie Esnault

**Affiliations:** ^1^ Hygiene and Quality of Poultry and Pig Products Unit, Ploufragan/Plouzané Laboratory ANSES, University of Bretagne‐Loire Ploufragan France

**Keywords:** genotyping, MLVA, PFGE, pig, slaughterhouse, *Yersinia enterocolitica*

## Abstract

The pig is one of the main reservoirs of *Yersinia enterocolitica* strains pathogenic to humans. A description of the *Y. enterocolitica* population in this reservoir, and accurate discriminatory techniques for typing isolates are needed for prevention, outbreak investigation, and surveillance. This study investigates the genetic diversity of pathogenic *Y. enterocolitica* isolates obtained from pig tonsils in a French pig slaughterhouse in 2009 (S1) and 2010 (S2). The use of Pulsed‐Field Gel Electrophoresis (PFGE) and MLVA as typing techniques was also compared and evaluated. First, a total of 167 isolates (12 of biotype 3 recovered during S1, and 155 of biotype 4 recovered during S1 and S2) were typed by PFGE using the XbaI enzyme. MLVA was then tested on all the biotype 3 isolates in addition to 70 selected biotype 4 isolates recovered over the 2 years. PFGE generated two specific XbaI‐PFGE profiles for biotype 3 isolates. Nine XbaI profiles were obtained for biotype 4, with a higher diversity (ID = 0.599) than biotype 3 (ID = 0.167). Two out of the nine XbaI profiles were reported during both surveys and at different months. MLVA improved the differentiation between isolates; the index of diversity reached 0.621 and 0.958, respectively, for biotype 3 (three MLVA types) and biotype 4 (32 MLVA types). The MLVA types for biotype 4 differed over the two surveys, but some isolates with different MLVA types were genetically closely related. This study provides an initial evaluation of the genetic diversity of *Y. enterocolitica* strains isolated from pigs in France. We show that some PFGE profiles are maintained in the pig production sector, and, through MLVA, that part of the *Y. enterocolitica* population remained genetically close over the two years. MLVA proved its effectiveness as a tool for investigating pathogenic *Y. enterocolitica* strains isolated from pigs.

## 1 INTRODUCTION

Yersiniosis is the third most frequently reported foodborne bacterial zoonosis after campylobacteriosis and salmonellosis, with an incidence of 1.82 cases per 100,000 European Union inhabitants in 2016 (EFSA & ECDC, 2017). Recently, Van Cauteren et al. (2017) estimated the annual number of foodborne yersiniosis cases in France at 21,330 with a credibility interval of ICr90% [10,799–49,477] for the 2008–2013 period. Among the *Yersinia enterocolitica* species isolated in France from humans, bioserotype 4/O:3 was by far the most frequent (71.1%), followed by bioserotype 2/O:9 (25.4%) and bioserotype 3/O:5,27 (1.8%) (Le Guern, Martin, Savin, & Carniel, 2016).

Pigs are considered to be the largest reservoir of pathogenic *Y. enterocolitica* strains. The bacteria can be isolated from feces (Van Damme, Vanantwerpen, Berkvens, & Zutter, 2014), tonsils (Fondrevez et al., 2014; Rahikainen Ibañez et al., 2016), and carcasses during slaughter (Van Damme et al., 2015).

Biotyping is used to evaluate the level of pathogenicity of *Y. enterocolitica* strains isolated from pigs, but it is insufficient to describe the diversity of pathogenic strains. It is necessary to characterize these strains more precisely within each biotype for the purposes of risk prevention, efficient outbreak investigation, and surveillance.

Different molecular typing techniques have been developed to more accurately describe *Y. enterocolitica* populations of porcine or human origin. The most commonly used of these techniques is Pulsed‐Field Gel Electrophoresis (PFGE). Many studies have used this typing technique, and various restriction enzymes have been tested to generate digestion profiles. Many studies, for example, have used NotI and XbaI as restriction enzymes (Bonardi et al., 2014; Falcão, Falcão, Pitondo‐Silva, Malaspina, & Brocchi, 2006; Fredriksson‐Ahomaa, Stolle, & Stephan, 2007). However, limited diversity among biotypes 4 and 3 was observed at pig slaughterhouse level even between strains of different geographical origins, like Germany, Finland, New Zealand, and China (Fredriksson‐Ahomaa et al., 2003; Gilpin et al., 2014; Liang et al., 2012). Another increasingly popular technique used to type *Y. enterocolitica* is the Multi‐locus variable number tandem repeat analysis (MLVA). Several studies have reported a higher discriminatory power with this technique (Gierczynski, Golubov, Neubauer, Pham, & Rakin, 2007; Sihvonen et al., 2011).

In our study, we tested two typing techniques—PFGE and MLVA—on a collection of *Y. enterocolitica* isolates of porcine origin isolated in a French slaughterhouse during two consecutive years. The aim was to evaluate the effectiveness of these two typing techniques to assess the diversity of the isolates and to evaluate their variation within the slaughterhouse over two years. Our study is the first one to describe the *Y. enterocolitica* population by both PFGE and MLVA in different years at the same slaughterhouse.

## 2 MATERIAL AND METHODS

### 2.1 *Yersinia enterocolitica* isolates

The *Y. enterocolitica* isolates considered in this study were collected during two surveys in the same French pig slaughterhouse. The latter is one of the largest slaughterhouses in France, with more than 1.5 million pigs slaughtered per year. The pigs slaughtered there have been supplied by the same farmers for many years.

The first survey (S1) was held from January to March 2009 (Fondrevez et al., 2010). This survey found 132 positive pigs (14.6%) out of the 900 pigs sampled in this slaughterhouse. The second survey (S2) was held from March 2010 to February 2011 (Fondrevez et al., 2014). This survey found 33 positive pigs (16.5%) out of the 200 pigs sampled in this slaughterhouse. The 200 pigs were sampled over 3 months (March, August, and November 2010) during this 1‐year survey (S2). We checked that the percentage of positive pigs in this slaughterhouse did not significantly change between the two surveys (*χ* ^2^ test, *p* = 0.511).

All the isolates were from pig tonsil swabs using the same bacteriological method as described previously (Fondrevez et al., 2014). Biochemical assays were used to biotype the *Y. enterocolitica* isolates and were carried out as per the ISO10273, 2003 method. One isolate of each biotype detected from a given swab was selected for this study. Consequently, a total of 167 isolates were selected for further typing analysis.

### 2.2 Pulsed‐field gel electrophoresis

Pulsed‐Field Gel Electrophoresis was carried out on all 167 *Y. enterocolitica* isolates included in this study. The *Salmonella* Braenderup H9812 strain was used as a reference size marker strain to allow comparison of the PFGE profiles from different gels.

Strains were sub‐cultured on Plate Count Agar (PCA) at 30°C for 24 hr. The culture was suspended in TE buffer (0.01 M Tris‐EDTA buffer, pH 8.0) and adjusted to an optical density (600 nm) of 1.5.

This suspension was then mixed with 1% agarose to make the plugs, which were incubated for 48 hr at 50°C in a lysis solution (Na_2_EDTA 0.5 M, pH9, *N*‐lauryl‐Sarcosyl 1%, proteinase K 1 mg/ml), and finally washed five times with TE buffer.

DNA was thereafter digested with 40 U of XbaI restriction enzyme (Roche, Boulogne‐Billancourt, France) for 4 hr at 37°C. The electrophoresis conditions had an initial switch time of 1.5 s, with a final switch time of 18.0 s for 25 hr at 6.6 V. The gels were stained with GelRed TM Nucleic Acid (Biotium) and the restriction fragments were visualized under ultraviolet light. Tiff images from the GEL Doc 1000 Imaging System (Bio‐Rad, Hercules, CA, USA) were imported into the ANSES laboratory’s database on the PFGE patterns of *Y. enterocolitica* strains. Electrophoretic patterns were compared using BioNumerics® (Applied Maths, Sint‐Martens‐Latem, Belgium; version 7.6). Similarities between profiles were determined by calculating the Dice correlation coefficient—based on band positions—with a maximum position tolerance of 1% on the active zones (8.5%–96.5%). A dendrogram was constructed in order to reflect the similarities between strains in the matrix. Strains were clustered by the unweighted pair group method using the arithmetic mean (UPGMA) (Struelens, 1996). Simpson’s index was determined as described by Hunter and Gaston (1988) to assess population diversity.

### 2.3 Multi‐locus variable number tandem repeat analysis (MLVA)

MLVA was used to type 82 isolates. These included the 12 biotype 3 isolates (S1 survey), 37 of the 122 biotype 4 isolates obtained in 2009 (S1 survey) and the 33 biotype 4 isolates obtained in 2010 (S2 survey). The 37 biotype 4 isolates were selected according to their XbaI‐PFGE profiles and their prevalence in 2009. Respectively, 5, 19, 4, 4, 2, and 3 isolates with profiles X02, X03, X04, X05, X09, and X11 were selected. MLVA was performed using the six primers—V2A, V4, V5, V6, V7, and V9—described by Gierczynski et al. (2007). The six VNTR loci were amplified in two distinct multiplex PCRs according to Sihvonen et al. (2011). The first one amplified VNTRs V2A, V4, and V6 with the forward primers labeled, respectively, by 6‐FAM, Cy3, and HEX fluorescent dyes. The second amplified VNTRs V5, V7, and V9 with the forward primers labeled, respectively, by 6‐FAM, HEX, and Cy3 fluorescent dyes. All the labeled and unlabeled primers were purchased from Sigma‐Aldrich. The multiplex PCRs were performed using a QIAGEN Multiplex PCR kit (Qiagen, Hilden, Germany) according to the manufacturer’s instructions in a total volume of 25 μl. The PCR conditions were the same as those described by Sihvonen et al. (2011). The two PCR products of each strain were diluted to 1/100 in sterile water, and run separately using capillary electrophoresis with an ABI 3130 DNA analyzer (Applied Biosystems, Foster City, CA, USA) with D (DS‐30) fragment analysis chemistry according to the manufacturer’s instructions. The Geneflo™ 625 ROX labeled (EurX, Gdańsk, Poland) was used as an internal size standard. Electrophoretic patterns were analyzed using BioNumerics 7.6 software (Applied Maths). To determine the correspondence between the allele size measured and the number of repeats, representative amplicons for all six VNTRs used in this study were subjected to sequence analysis using a BigDye Terminator v3.1 cycle sequencing kit (Applied Biosystems) with an ABI 3130 DNA analyzer (Applied Biosystems). An MLVA type was given for each combination of the six VNTRs and attributed to the isolates (Supporting Information Table S1). Simpson’s index (ID) was determined to assess the diversity of the populations and the discriminatory power of each VNTR locus. A standard minimum spanning tree generated under BioNumerics 7.6 using the single and double locus variance priority rules was used to visualize the relationships between biotype 4 isolates.

### 2.4 Comparison of the methods

The methods were compared using 80 isolates, including the 12 biotype 3 isolates (S1 survey), the 33 biotype 4 isolates obtained in 2010 (S2 survey) and 37 out of the 122 biotype 4 isolates obtained in 2009 (S1 survey). To avoid a sampling effect, the 37 biotype 4 isolates out of the 122 isolates available were selected according to two criteria: their XbaI‐PFGE profiles and their prevalence in the population recovered in 2009. We validated this sampling because the population represented by the 37 isolates was not significantly different from the population represented by the 122 isolates (Fisher’s test, *p* = 0.179).

Pulsed‐Field Gel Electrophoresis and MLVA were compared according to two criteria: their discriminatory power estimated using Simpson’s index (ID), and their concordance. This congruence was assessed using the adjusted Rand index (AR) which measures the overall agreement between two typing techniques (Hubert & Arabie, 1985) and the adjusted Wallace index (AW) which assesses the directional agreement by separately evaluating the concordance when each technique is used first (Severiano, Pinto, Ramirez, & Carrico, 2011). The adjusted Rand and the adjusted Wallace coefficients were determined using website https://www.comparingpartitions.info/(Carrico et al., 2006).

## 3 RESULTS

### 3.1 Distribution of the isolates

The 167 isolates were distributed among two biotypes, with biotype 4 being the most prevalent (92.81% of the isolates). The other 12 isolates (7.18%) belonged to biotype 3, and were only recovered from the S1 survey. Interestingly, out of the 132 pigs detected positive for *Y. enterocolitica*, two carried both a biotype 4 and a biotype 3 isolate. The other 130 pigs were detected positive for only one biotype, either biotype 4 or biotype 3. No biotype 3 isolates were recovered from the S2 survey carried out on 200 pigs. This distribution of the biotypes from isolates in both surveys was not significantly different (Fisher’s test; *p* = 0.126).

### 3.2 Diversity using PFGE

A total of 11 XbaI‐PFGE profiles were obtained from the 167 isolates and were coded X01–X11 (Table 1). Simpson’s index (ID) of diversity for all isolates was equal to 0.656 [0.583–0.729]. Biotype 3 could be differentiated from biotype 4 using the XbaI enzyme as a restriction enzyme for PFGE.

**Table 1:** Distribution of the 167 *Yersinia enterocolitica* isolates according to their XbaI‐PFGE profile

XbaI profile	BT3 (S1)	BT4 (S1)	BT4 (S2)	Total isolates
X01	11	—	—	11
X02	—	12	—	12
X03	—	87	6	93
X04	—	10	15	25
X05	—	5	—	5
X06	—	—	1	1
X07	—	—	1	1
X08	—	—	10	10
X09	—	3	—	3
X10	1	—	—	1
X11	—	5	—	5
Total isolates	12	122	33	167
ID	0.167	0.475	0.688	0.656
95% CI	0‐0.430	0.370‐0.580	0.598‐0.777	0.583‐0.729

Note

BT: biotype; CI: confidence interval; ID: index of diversity; S1: first survey 2009; S2: second survey 2010–2011.

**Figure 1:**
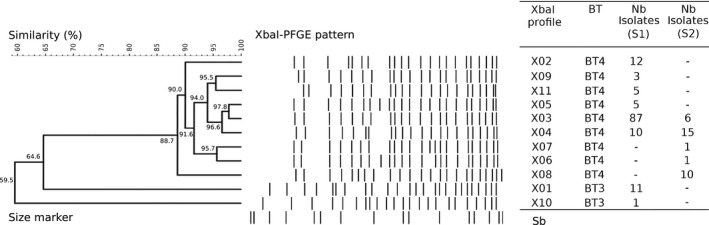
Dendrogram of XbaI‐PFGE profiles for *Yersinia enterocolitica* isolates from surveys S1 and S2 (167). BT: biotype; Nb isolates: number of isolates; S1: first survey 2009; S2: second survey 2010–2011; Sb: *Salmonella* Braenderup H9812

The 12 biotype 3 isolates were distributed only among two XbaI‐PFGE profiles: X01 (11) and X10 (1). There was therefore very little diversity (ID = 0.167 95% CI [0–0.430]). These profiles were only observed for this biotype and were genetically distant from biotype 4 on the dendrogram (<64.5% of genetic similarity; Figure 1). In addition, the X10 profile was very different from the X01 profile (59.5% of genetic similarity).

The 155 biotype 4 isolates were distributed among nine XbaI‐PFGE profiles. The diversity was higher for this biotype (ID = 0.605 95% CI [0.525–0.685]) than for biotype 3. Six of the XbaI‐PFGE profiles were observed during S1 and five during S2 (Table 1). The diversity differed from one survey to another: it was lower for S1 (ID = 0.475 95% CI [0.370–0.580]) than S2 (ID = 0.688 95% CI [0.598–0.777]).

The biotype 4 isolates were closely related, sharing at least 88.7% of genetic similarity on the dendrogram (Figure 1). Of the nine XbaI‐PFGE profiles, X03 was the most prevalent (particularly during S1; Table 1); 60.0% of all the isolates (93/155) had this profile, followed by X04, with 16.13% of the isolates (25/155). These two XbaI‐PFGE profiles, X03 and X04, were the only ones observed in both surveys; the others were observed only during either the first survey (X02, X05, X09 and X11) or the second survey (X06, X07 and X08).

X03 was found over all three months in 2009 (S1) and 2010 (S2), while X04 was found in February and March in 2009, then August and November in 2010. The presence of these profiles is therefore not associated with the season.

### 3.3 Diversity using MLVA and comparison with PFGE

A total of 82 isolates were typed by MLVA; the 12 biotype 3 isolates (S1 survey), and 70 biotype 4 isolates (37 from the S1 survey, and 33 from the S2 survey). A PCR product was obtained for all six VNTR loci (V2A, V4, V5, V6, V7, V9) except for one biotype 3 strain for which loci V6 and V9 were not amplified (Supporting Information Table S1). Analysis of loci V2A, V4, V5, V6, V7, and V9 separately showed that Simpson’s index (ID) of diversity varied from 0.804 to 0.900 (Table 2). Locus V4 had the lowest discriminatory power (ID = 0.804 95% CI [0.771‐0.837]) and loci V2A and V5 the highest discriminatory power (ID = 0.900 95% CI [0.875‐0.925]; ID = 0.879 95% CI [0.846‐0.912]). A total of 35 MLVA types were obtained by combining the six VNTR loci from the 82 isolates. The MLVA types differed by two to six VNTR loci. The combinations were coded M01 to M35 (Table 3), with one prevalent MLVA type (M26) grouping 11 isolates. Simpson’s index (ID) of diversity for all isolates was equal to 0.962 95% CI [0.945–0.979].

**Table 2:** Discriminatory power of the six VNTR loci

locus VNTR	V2A	V4	V5	V6	V7	V9
ID	0.900	0.804	0.879	0.861	0.850	0.847
95% CI	0.875‐0.925	0.771‐0.837	0.846‐0.912	0.832‐0.890	0.813‐0.887	0.818‐0.876

Note

CI: confidence interval; ID: index of diversity.

**Table 3:** Distribution of the 82 *Yersinia enterocolitica* isolates according to their MLVA and XbaI‐PFGE profile

MLVA type	XbaI‐PFGE profile													Total isolates
	BT3 (S1)		BT4 (S1)						BT4 (S2)					
	X01	X10	X02	X03	X04	X05	X09	X11	X03	X04	X06	X07	X08	
M01		1												1
M02								1						1
M03								1						1
M04					3									3
M05	5													5
M06								1						1
M07				1										1
M08			4											4
M09			1											1
M10							2							2
M11	6													6
M12				1										1
M13					1									1
M14				3										3
M15				2										2
M16				2										2
M17				1										1
M18						2								2
M19				1										1
M20				3										3
M21						2								2
M22				1										1
M23				2										2
M24				1										1
M25				1										1
M26										11				11
M27										2				2
M28										2				2
M29									1					1
M30													6	6
M31											1	1		2
M32									1					1
M33													4	4
M34									2					2
M35									2					2
Total isolates	11	1	5	19	4	4	2	3	6	15	1	1	10	82
ID	0.621		0.968						0.847					0.962
95% CI	0.492‐0.751		0.951‐0.986						0.764‐0.929					0.945‐0.979

Note

BT: biotype; CI: confidence interval; ID: index of diversity; MLVA types: M01–M35; S1: first survey 2009; S2: second survey 2010–2011; XbaI‐PFGE profiles: X01–X11.

The 12 biotype 3 isolates were distributed among three MLVA types: M01 (the isolate with XbaI‐PFGE profile X10), M05 (5) and M11 (6) (these 11 isolates having XbaI‐PFGE profile X01). These MLVA types were only observed for this biotype. The diversity for this biotype increased significantly with this typing technique (ID = 0.621 95% CI [0.492–0.751]) compared to XbaI‐PFGE (ID = 0.167 95% CI [0–0.430]).

**Figure 2:**
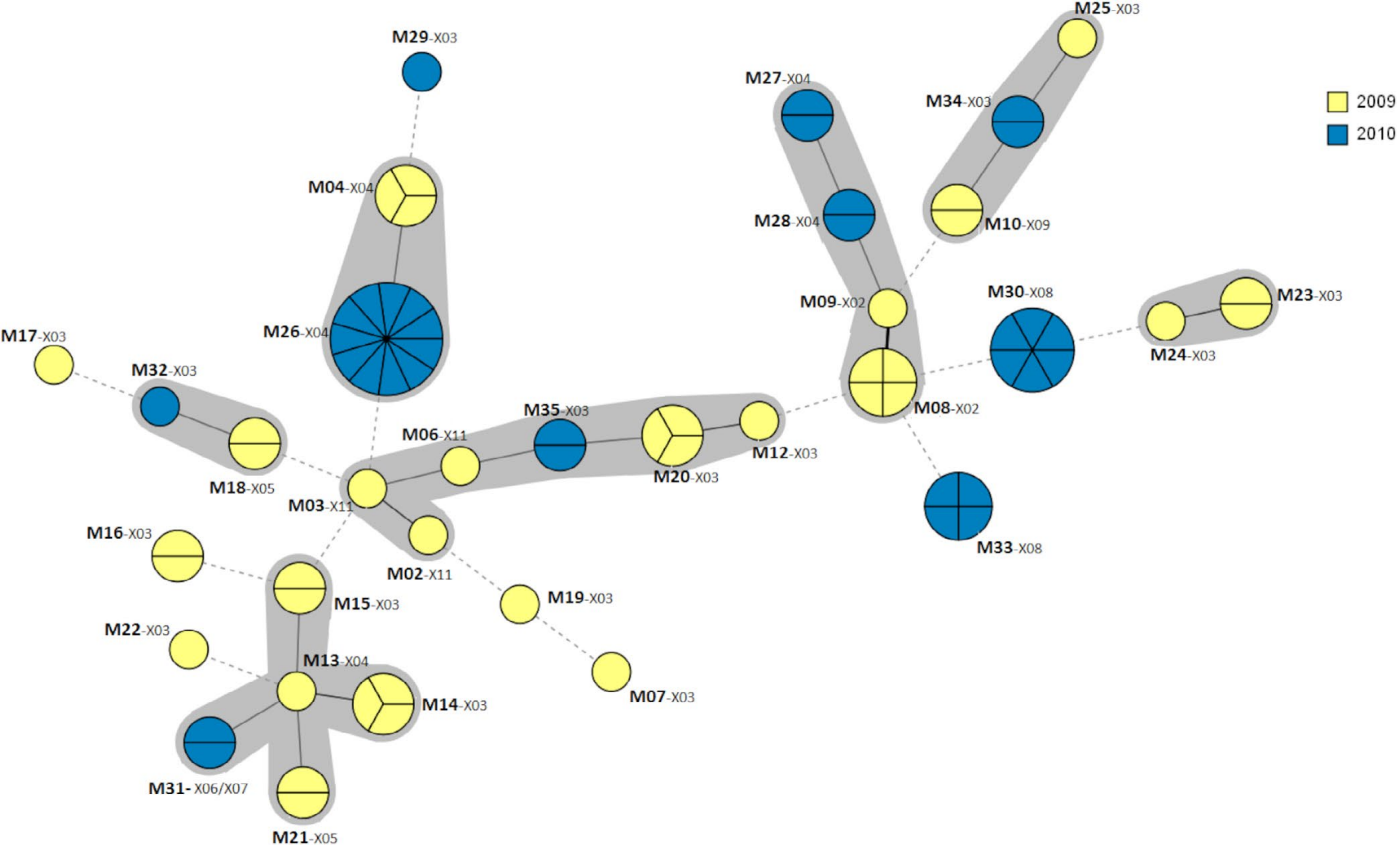
Distribution of the biotype 4 isolates (*n* = 70) in a minimum spanning tree according to their MLVA types (M01 to M35). To visualize the relationships between isolates, a standard minimum spanning tree (MST) was generated using BioNumerics software (ver. 7.6) with a categorical coefficient of similarity and single and double locus variance priority rules. Each circle represents a different MLVA type, its size being proportional to the number of strains belonging to that MLVA type. Branch thickness indicates how many loci are different in the MLVA types of the connected circles. Thick solid lines connect nodes that differ by one MLVA locus, thin solid lines connect nodes that differ by two or three MLVA loci and dashed lines connect nodes that differ by more than three MLVA loci. The halo surrounding the MLVA types groups together types belonging to the same complex. MLVA complexes were assigned if two neighboring types did not differ by more than three VNTR loci and if at least two types fulfilled this criterion. The XbaI‐PFGE profiles (X01–X11) are also indicated near the corresponding MLVA type (M01–M35)

The 70 biotype 4 isolates were distributed among 32 MLVA types (Table 3). The diversity was higher for this biotype (ID = 0.958 95% CI [0.935–0.981]) than for biotype 3, and was shown by this typing technique to be significantly higher than when XbaI‐PFGE was used (ID = 0.605 95% CI [0.525–0.685]). A single MLVA type (M31) grouped isolates with different XbaI‐PFGE profiles (X06 and X07). Twenty‐five MLVA types were observed during S1, and ten during S2 (Table 3); furthermore, there were no MLVA types common to both surveys, unlike with XbaI‐PFGE. The diversity differed from one survey to another; there was a greater diversity for S1 (ID = 0.968 95% CI [0.951–0.986]) than for S2 (ID = 0.847 95% CI [0.764–0.929]). Although the MLVA types were different from one survey to another, their distribution in a minimum spanning tree (MST) showed no clustering on a yearly basis (Figure 2). The MLVA types connected with a link corresponding to a difference of less than three MLVA loci were grouped together into a complex. Of the 35 MLVA types, eight did not belong to any complex. The other 27 MLVA types clustered into seven complexes. One complex grouped together isolates from 2009, and six complexes included isolates from 2009 and 2010.

The concordance of the two genotyping techniques was assessed by calculating the Adjusted Rand (AR) and Adjusted Wallace (AW) indexes. The Adjusted Rand index, which assesses the overall accordance between two techniques, revealed no congruence between the two techniques (0.330). Using the Adjusted Wallace index (AW), we assessed the directional agreement between the techniques (Severiano et al., 2011). Therefore, when MLVA was used before PFGE, the congruence with PFGE reached 98.1% (AW_MLVA→PFGE_ = 0.981 [0.968–0.994]), while the agreement decreased to 19.9% when PFGE was used first (AW_PFGE→MLVA_ = 0.199 [0.121–0.277]). Population modeling using MST according to the isolates’ MLVA types made the lack of correlation between both typing techniques even more obvious (Figure 2).

## 4 DISCUSSION

This study is the first to describe the *Y. enterocolitica* population among pigs slaughtered at the same slaughterhouse in two consecutive years and using two typing techniques. It highlights the relevance of the typing technique for monitoring the variation of this population over time.

Most of the isolates considered in this study were of biotype 4 (92.81%). The predominance of biotype 4, which is pathogenic to humans, is in accordance with many previous studies on pigs (Bonardi et al., 2014; Martinez et al., 2011; Martins et al., 2018; Rahikainen Ibañez et al., 2016; Van Damme, Habib, & Zutter, 2010). Biotypes 2 and 3 were also found in the slaughtered pigs examined. These biotypes are generally less common, with a prevalence below 10% (Fredriksson‐Ahomaa et al., 2007; Martinez et al., 2011; Poljak et al., 2010). Although no biotype 2 isolates were detected in our study, some biotype 3 isolates were recovered during survey S1 in 2009. Statistical analysis indicated that the percentage of positive pigs and the distribution of isolates according to biotype did not differ significantly from one survey to another. The absence of biotype 3 in the second survey could be explained by the smaller number of pigs sampled (200) compared to the first survey (900).

To evaluate the genetic diversity of *Y. enterocolitica* isolated at slaughterhouse level in both surveys, we first typed the isolates by PFGE with restriction enzyme XbaI. The PFGE method has been widely used with different restriction enzymes to distinguish isolates of *Y. enterocolitica*. Isolates of biotypes 3 and 4 were differentiated using XbaI by Buchrieser, Weagant, and Kaspar (1994) and Najdenski, Iteman, and Carniel (1994). In our study, we also observed only two XbaI profiles among the 12 biotype 3 isolates. These profiles, X01 and X10, were only found for biotype 3 and clearly differed from biotype 4 profiles, with a genetic similarity less than 64.5%. A different finding was observed by Gilpin et al. (2014). When using ApaI or NotI enzymes alone or in combination, similar profiles were identified for biotypes 3, 2 and 4.

Our study revealed a low diversity of biotype 3 isolates(ID: 0.167). This lack of diversity may be explained either by the fact that we had only a few isolates belonging to this biotype, or because biotype 3 is generally less diverse. This low diversity for biotype 3 was previously observed by Liang et al. (2012) (ID of 0.522) and by Gilpin et al. (2014) (ID of 0.440) when they typed isolates using different restriction enzymes.

In those studies as well as in ours the majority of biotype 3 isolates belonged to one common profile shared by, respectively, 75.0%; 53.1%, and 91.7% of the isolates (Gilpin et al., 2014; Liang et al., 2012; this study). The choice of the enzyme or combination of enzymes did not seem to significantly improve the power of PFGE to discriminate biotype 3 isolates. Moreover, the 439 strains tested in China by Liang et al. (2012) had profiles with 79% of similarity. This supports the idea that PFGE encountered little genetic diversity among biotype 3 isolates and failed to subtype this biotype.

Interestingly, the two XbaI profiles recovered in our study shared only 59.5% of similarity. This low similarity may indicate that profiles were genetically distant from each other. Since only one isolate with the X01 profile had been recovered from the 12 biotype 3 isolates, we speculated that there may have been a recent emergence or disappearance of isolates having the X01 profile to the benefit of isolates having the X10 profile. Another hypothesis may be that X01 isolates were not as well‐adapted as X10 isolates, and therefore remained at a low level of prevalence. In order to conclude on the biotype 3 isolates’ genetic diversity and on the usefulness of PFGE for their typing, another typing method needed to be tested.

The 155 biotype 4 isolates in our study were grouped into nine XbaI profiles, giving an index of diversity of 0.605 over the two surveys. Compared to biotype 3 isolates, biotype 4 isolates were better subtyped with the PFGE method. Also always superior to 0.500, the value of discriminatory index for biotype 4 may vary according to studies. The diversity of the biotype 4 population was similar for Fredriksson‐Ahomaa, Korte, and Korkeala (2000) when they used the XbaI enzyme alone (ID = 0.594). Using NotI, three other studies obtained, respectively, an index of diversity of 0.564, 0.692 and 0.840 (Fredriksson‐Ahomaa et al., 2000; Gilpin et al., 2014; Sihvonen et al., 2011). Since the same enzyme was used the difference in the ID should have reflected the genetic variability between the set of strains studied.

The PFGE discriminating power increased from 0.740 to 0.870 and 0.930 when Fredriksson‐Ahomaa, Autio, and Korkeala (1999) used, respectively, NotI alone, NotI and ApaI or NotI, ApaI and XhoI to type biotype 4 strains. The use of a combination of enzymes may then increase the discriminating power of PFGE when biotype 4 isolates are studied. The choice of enzymes or their combination could therefore be an important parameter to take into account for an optimal discrimination of biotype 4 isolates. In our study, we noted that the biotype 4 population in the first survey (S1) was less diverse than that of the second survey (S2), despite the number of isolates tested during S1 being much larger (122 vs. 33). This may be due to the fact that samples from the first survey were concentrated over three months in 2009, while those from the second survey were spread out more over time, increasing the probability for the 33 isolates to have different profiles.

Although not identical, the profiles of biotype 4 isolates from both surveys exhibited only minor differences. The isolates with a high degree of similarity (88.7%) were then grouped, independently of the year of survey. The marked homogeneity of biotype 4 isolates had previously been observed with different sets of isolates and different enzymes (Bonardi et al., 2014; Falcão et al., 2006; Filetici, Anastasio, Pourshaban, & Fantasia, 2000).

In our study, PFGE divided biotype 4 isolates into two major groups representing 71% of all the tested isolates. Several analyses of the biotype 4 population with PFGE revealed the presence of one or two dominating profiles (Bonardi et al., 2014; Fredriksson‐Ahomaa et al., 2000, 2007 ; Gilpin et al., 2014). These profiles represented, respectively, 58% to 77% of the population studied. When considering the pulsotype, the major profiles were closely related (96.5% of similarity). We can therefore hypothesize that they shared common characteristics which promoted their persistence in the slaughtered pigs.

Moreover, the number and type of PFGE profiles differed from one year to the other. Thus, in our study, four XbaI‐PFGE profiles identified in the first survey were no longer observed in the second survey, whereas three new XbaI‐PFGE profiles were identified in the second survey. Therefore, the population of *Y. enterocolitica* must have varied over time, although XbaI profiles displayed minor deviations and were genetically closely related (sharing at least 88.7% of genetic similarity).

The genetic variation of *Y. enterocolitica* appeared to be uncorrelated to the year of isolation. Nevertheless, we observed that 76. % of the biotype 4 isolates 4 belonged to two XbaI‐PFGE profiles (X03 and X04) founded throughout the two surveys and at different months. Because the different profiles may compete and suffer mutual interference, the presence of the same profile over the years can be considered of interest. This situation had already been described previously. Fredriksson‐Ahomaa, Meyer, Bonke, Stuber, and Wacheck (2010) recovered six NotI‐ApaI‐XhoI genotypes from the pigs slaughtered in the same slaughterhouse over more than one year. These genotypes came from 14 out of the 27 farms found positive to *Y. enterocolitica*. Another study concerning a retrospective analysis of clinical biotype 4 strains isolated between 2008 and 2010 indicated that some profiles persisted over several years (Martin, Cabanel, Lesoille, Menard, & Carniel, 2015). These observations suggest either that some biotype 4 genotypes were widely distributed and persisted for years, or that the PFGE method could not discriminate all strains. A clear predominance of major profiles may lead to an incorrect attribution of isolates, especially in case of possible outbreaks. As a matter of fact, the relationship between an isolate and an outbreak cannot be established if the isolate belongs to one of the major profiles.

In our study, the genetic variation of *Y. enterocolitica* biotypes 3 and 4 appeared to be quite limited when using the PFGE typing technique. As several studies demonstrated MLVA's high discriminatory power (Gierczynski et al., 2007; Sihvonen et al., 2011) when applied to *Y. enterocolitica* from different sources. Alakurtti et al. (2016) reported that the discriminatory power of the loci varied from one country to another. Loci V4 and V9 are often reported to have a low discriminatory power compared to the other loci (Alakurtti et al., 2016; Sihvonen et al., 2011; Wang et al., 2012). In these studies, the discriminatory power value was between 0 and 0.60 for one of the two loci. Compared to MLVA data obtained from other countries, the present study indicated that all six VNTR loci showed a high discriminatory power with a value over 0.80. Nevertheless, by considering the IC95%, locus V4 was statistically less discriminatory than loci V2A and V5.

MLVA has been reported to correctly differentiate biotype 4 from biotype 2 strains (Sihvonen et al., 2011), and biotype 3 (serotype O:3) from biotype 2 (serotype O:9) strains (Wang et al., 2012). No MLVA types were observed to be common to both biotype 4 and 3 isolates in our study. Using this technique, we reinforced the evidence that MLVA can successfully distinguish biotype 4 and biotype 3 isolates. Moreover, we found a greater genetic diversity when typing isolates with MLVA compared to XbaI‐PFGE, whatever the biotype. For biotype 4, we obtained 38 MLVA types with a diversity index of 0.964, and only nine profiles with PFGE. This result is in accordance with those of Sihvonen et al. (2011); with the same MLVA scheme (V2A, V4, V5, V6, V7, and V9) they obtained a Simpson's index of 0.999. Concerning biotype 3 isolates, we also obtained three MLVA profiles versus two PFGE profiles. Our results are in agreement with those obtained by Wang et al. (2012), who showed that MLVA improved the distinction between strains within biotype 3 when using MLVA instead of NotI‐PFGE.

MLVA additionally divided the main PFGE types into several subtypes. Because of its high discriminatory power, MLVA showed to be a helpful tool for discriminating isolates difficult to differentiate by PFGE but epidemiologically unrelated. Sihvonen et al. (2011) reported the usefulness of MLVA to support epidemiological data concerning an outbreak due to a 4/O:3 *Y. enterocolitica* infection. During the investigation, MLVA typing was used to make a distinction between strains isolated from patients and those that were epidemiologically unrelated although sharing the same PFGE profile. The use of MLVA to investigate human yersiniosis in a French region allowed strains belonging to a major PFGE profile to be subtyped (Martin et al., 2015) and arguments about connections between the cases to be put forward. Because MLVA revealed a remarkably high genetic diversity of the pathogenic strains, the authors refuted the hypothesis of a single source of contamination or the expansion of a specific clone.

When using MLVA, we noted that the biotype 4 population found in the first survey was more diverse than that of the second survey, despite a similar number of tested isolates (37 vs. 33). This finding may be explained by the fact that for MLVA we extracted isolates from the first survey based on their PFGE profile. This selection was not made on the isolates from the second survey. Nevertheless, this is unlikely since the prevalence of the PFGE profiles was taken into account when selecting the 37 isolates and provided a sub‐population representative of the total population of isolates recovered in 2009.

During recent years, there has been a shift from PFGE to MLVA genotyping techniques for *Y. enterocolitica*. Some laboratories involved in the national surveillance of yersiniosis have used this technique to type strains isolated during outbreaks (MacDonald et al., 2016, 2012; Martin et al., 2015). MLVA was used because of its good discrimination of pathogenic *Y. entrocolitica,* especially within biotypes 4 and 2, the major biotypes encountered in human yersiniosis (Drummond et al., 2012). A comparison of the two methods indicated that MLVA may supplant PFGE because of its typeability, reproducibility and feasibility in addition to its typing properties. MLVA had a higher discriminatory power and could predict the partition obtained with PFGE (AW: 98.1). On the contrary, when using PFGE first, the use of MLVA as the second typing method could provide further information, improving the discrimination of isolates (AW: 19.9). Nevertheless, no congruence between the two partition results (AR: 0.330) was observed. Biotype 4 population MST modeling using MLVA clustered isolates irrespective of their PFGE profile. The gain in discriminatory power may not in fact be correlated with a gain in confidence about the biological interpretation of the results. To address this issue, the relevance of MLVA in terms of stability has been investigated in vitro and in vivo. Gierczynski et al. (2007) showed that the MLVA type remained unchanged after 20 serial passages of a strain in vitro. In vivo, the same MLVA type was recovered from humans after repeated isolations from one or more patients with yersiniosis (Gierczynski et al., 2007; Sihvonen et al., 2011) as well as in pigs on farms when sampled at a 6‐month interval (Saraka et al., 2017). Thus, like PFGE profiles, MLVA types are likely to remain stable and survive unchanged for long periods. In our study, also genetically closely related, no isolates with the same MLVA type were recovered from both surveys. Unfortunately, we have no data concerning the farm from which the pigs came. The presence of different MLVA profiles on pig tonsils from one survey to the other may be explained by the fact that the sampled pigs came from different farms during the two surveys, or that they were contaminated with strains from other pig batches during transport, lairage, and slaughter. Tonsils are among the first organs to be contaminated and they have a high concentration of *Y. enterocolitica* when contaminated (Van Damme et al., 2015).

Some isolates recovered in both years had the same PFGE profile and a genetically close MLVA type. Among the ten MLVA types described in 2009, eight belonged to complexes including isolates from 2009 and 2010, and three of them shared a PFGE profile common to isolates from 2010. Isolates having few genetic differences in terms of MLVA types may thus be isolated in each of the two years. This may suggest that porcine *Y. enterocolitica* population persisted for years and varied over time. Further studies should be investigated in order to conclude on a possible correlation between MLVA type variation and the relatedness of isolates.

## 5 CONCLUSION

This study provides an initial evaluation of the genetic diversity of *Y. enterocolitica* strains isolated from pigs in France. The biotype 4 population is genetically more heterogeneous than the biotype 3 population. With PFGE, we showed that some profiles were maintained in the pig production sector due to the presence of two XbaI‐PFGE profiles in two consecutive years. With MLVA, we not only improved the differentiation between isolates, but also showed that clones recovered during both years may be genetically closely related. In addition, our study showed that MLVA successfully discriminated biotype 4 from biotype 3 isolates. MLVA, in combination or not with PFGE, has proved its effectiveness as a tool for investigating pathogenic *Y. enterocolitica* strains isolated from pigs and assessing the genetic diversity of this foodborne pathogen. Because typeability, reproducibility, and discriminatory power are key features in the evaluation of an epidemiological typing system, MLVA is a promising tool. Nevertheless, further studies are needed to improve our knowledge on how to establish a clear relationship between MLVA profiles and epidemiological data.

## CONFLICT OF INTEREST

The authors declare no conflicting interests.

## AUTHORS CONTRIBUTION

Conceived and designed the experiment: EE. Performed the experiment: PR, EH, EE. Analyzed the data: EE, PR. Critical discussion about the results: EE, PR, MD. Wrote the paper: EE, MD, PR. Final approval for the submitted documents: PR, EH, MD, EE. Funding: EE.

## ETHICS STATEMENT

The research was done on isolates collected from tonsil swabs on pig carcases. No ethical approval for this study was needed.

## Data Availability

Supporting Information Table S1 is included at the end of the manuscript in the section "Appendices". The authors adhere to all policies on sharing data and materials described in the guidelines for authors.
