# Supplementary material for: Diversity of Yersinia enterocolitica isolated from pigs in a French slaughterhouse over 2 years
Source: Microbiologyopen. 2018 Oct 22;8(6):e00751. doi: 10.1002/mbo3.751 (PMC6562139; doi:10.1002/mbo3.751)
Supplement: Supplementary file 1 [file MBO3-8-e00751-s001.docx]

**Appendices:**

**Table S1.** Correspondence between MLVA type and the number of repetitions for each locus

| **MLVA type** | **V2A-V4-V5-V6-V7-V9**  (number of repetitions of each locus) |
| --- | --- |
| M01 | 01-02-02-00-06-00 |
| M02 | 05-03-11-07-13-07 |
| M03 | 05-05-11-07-13-06 |
| M04 | 05-08-14-04-06-11 |
| M05 | 06-02-11-08-08-03 |
| M06 | 06-05-11-08-12-06 |
| M07 | 07-03-07-05-12-04 |
| M08 | 07-06-03-06-05-06 |
| M09 | 07-06-03-06-06-06 |
| M10 | 07-07-13-03-06-09 |
| M11 | 08-02-09-06-08-03 |
| M12 | 08-05-12-10-05-06 |
| M13 | 09-05-09-08-11-08 |
| M14 | 09-06-09-08-13-08 |
| M15 | 10-05-09-08-13-07 |
| M16 | 10-05-14-04-14-05 |
| M17 | 10-07-08-10-09-03 |
| M18 | 11-05-14-14-04-06 |
| M19 | 12-03-12-07-20-04 |
| M20 | 12-05-10-10-05-06 |
| M21 | 13-05-09-08-09-05 |
| M22 | 17-05-14-18-11-07 |
| M23 | 19-05-07-09-10-03 |
| M24 | 21-06-07-09-10-03 |
| M25 | 22-07-20-03-05-08 |
| M26 | 05-08-11-03-09-11 |
| M27 | 06-06-06-07-07-06 |
| M28 | 06-06-08-06-08-06 |
| M29 | 06-09-14-03-05-11 |
| M30 | 09-06-17-09-05-05 |
| M31 | 09-07-07-08-06-08 |
| M32 | 10-05-15-14-09-06 |
| M33 | 11-06-14-07-05-08 |
| M34 | 14-07-13-03-05-10 |
| M35 | 17-05-10-08-06-06 |
